# Identification of fungi in shotgun metagenomics datasets

**DOI:** 10.1371/journal.pone.0192898

**Published:** 2018-02-14

**Authors:** Paul D. Donovan, Gabriel Gonzalez, Desmond G. Higgins, Geraldine Butler, Kimihito Ito

**Affiliations:** 1 School of Biomedical and Biomolecular Science and UCD Conway Institute of Biomolecular and Biomedical Research, Conway Institute, University College Dublin, Belfield, Dublin, Ireland; 2 Division of Bioinformatics, Research Center for Zoonosis Control, Hokkaido University, Sapporo, Hokkaido, Japan; 3 School of Medicine and UCD Conway Institute of Biomolecular and Biomedical Research, University College Dublin, Belfield, Dublin, Ireland; 4 Global Station for Zoonosis Control, Global Institution for Collaborative Research and Education, Hokkaido University, Sapporo, Hokkaido, Japan; University of Minnesota, UNITED STATES

## Abstract

Metagenomics uses nucleic acid sequencing to characterize species diversity in different niches such as environmental biomes or the human microbiome. Most studies have used 16S rRNA amplicon sequencing to identify bacteria. However, the decreasing cost of sequencing has resulted in a gradual shift away from amplicon analyses and towards shotgun metagenomic sequencing. Shotgun metagenomic data can be used to identify a wide range of species, but have rarely been applied to fungal identification. Here, we develop a sequence classification pipeline, FindFungi, and use it to identify fungal sequences in public metagenome datasets. We focus primarily on animal metagenomes, especially those from pig and mouse microbiomes. We identified fungi in 39 of 70 datasets comprising 71 fungal species. At least 11 pathogenic species with zoonotic potential were identified, including *Candida tropicalis*. We identified *Pseudogymnoascus* species from 13 Antarctic soil samples initially analyzed for the presence of bacteria capable of degrading diesel oil. We also show that *Candida tropicalis* and *Candida loboi* are likely the same species. In addition, we identify several examples where contaminating DNA was erroneously included in fungal genome assemblies.

## Introduction

Fungi represent one of the major Kingdoms of the Eukaryotic domain of life. Some species are of great economic importance, providing antibiotics, fermenting foods such as beers and breads, and degrading cellulose. It is estimated that there are millions of fungal species, although only a small number have been characterized [1]. The lack of characterized species results from a number of factors, such as phenotypic diversity, genome plasticity, and the inability to culture the majority of species [2, 3].

In recent years, there has been a gradual shift from studying isolated species to studying their interactions in an environment that is more representative of their ecological niche. This shift is reflected in the increased use of nucleic acid sequencing directly from an environmental sample with no prior knowledge of the species that are present. The collection of microbial organisms that are found in any particular environment is known as the microbiota, whereas the microbiome refers to all genetic material in the microbiota, and metagenomics is the study of the genetic material within the microbiota [4]. The terms metagenome and microbiome are often used interchangeably.

The mycobiome is the fungal component of the microbiome. The term was first used in 2010, in reference to the human oral mycobiome [5]. The number of mycobiome publications has increased at an average rate of ~60% each year since 2012 (as of late 2017). Nevertheless, this area remains understudied compared to bacterial microbiomes [6]. Most published work has focused on the human [7, 8] or soil [9] mycobiome. However, several recent studies suggest that animals can carry potentially zoonotic fungi. For example, *Candida* species were discovered on ticks from a seabird colony in Ireland, in pigeon feces from Gran Canaria, and in bat droppings [10–12]. Animals could represent significant fungal reservoirs for human fungal infection. In addition, we often do not know the environmental reservoir of fungal microbes, and microbiome studies can greatly contribute to this field.

Two sequence-based methods are generally used to identify fungal species in a mycobiome. The most common is PCR amplification of internal transcribed spacer (ITS) regions of rRNA operons, in particular ITS2 between the 5.8S and 28S genes, followed by sequencing. ITS2 sequences are highly variable and have been adopted as the universal fungal barcode sequence for fungi [13]. Several pipelines have been developed to identify specific fungal species and calculate the frequency of each species from ITS data, including Plutof, Clotu, PIPITS, and CloVR-ITS [14–17]. BioMaS, Mothur and Qiime can be used with both bacterial and fungal amplicon reads [18–20].

The second approach identifies species from shotgun metagenomes. Most tools use custom-built databases, together with search algorithms such as BLAST, USEARCH and UBLAST, GhostX, and DIAMOND [21–24]. These tools identify the database sequence most similar to a read from a metagenome. Alternatively, algorithms such as KAIJU and Kraken assign reads to a lowest common ancestor (LCA) [25, 26]. KAIJU translates reads and compares them against a reference protein database, whereas Kraken compares nucleotide queries to a nucleotide database. Both KAIJU and Kraken are fast because they use exact k-mer matches, as opposed to slower alignment based approaches.

Some metagenomics databases implement their own pipelines to simultaneously host and analyze datasets. MG-RAST provides detailed graphical analyses of user-uploaded datasets using an incrementally updated pipeline [27], and has been used to identify fungi in grain dust from a swine facility [28]. However, the ability of the pipeline to detect eukaryotic DNA is based on comparing sequence reads to rDNA, ignoring all non-rDNA reads. The European Bioinformatics Institute also hosts a metagenomics database with an associated pipeline, called EBI Metagenomics [29]. EBI Metagenomics contains a large number (~16,000) of well-curated datasets, but only began identifying eukaryotic DNA following version 4.0 release (4^th^ September 2017). Less than 1% of the EBI Metagenomics datasets have been analyzed using pipeline v4.0, and like MG-RAST, only rDNA sequences are used. The Joint Genome Institute has developed IMG/M to facilitate the storage and analysis of genomics and metagenomics datasets [30]. These resources are in their infancy and are updated regularly, and likely represent the future for metagenomics dataset analyses.

Here, we describe FindFungi, a pipeline for identifying fungal species in shotgun metagenomics datasets, without relying on rDNA amplicons. We combine read identification using Kraken [26] with an analysis of read distribution across the target genome, which greatly reduces false positives. The method has high sensitivity and specificity. We use FindFungi to identify fungal species (including potential zoonotic fungi such as *Candida tropicalis*) in animal metagenomes. All code for FindFungi (version 0.23) is available on Github at https://github.com/GiantSpaceRobot/FindFungi-v0.23.

## Results and discussion

### Pipeline construction and testing

To find the best method for identifying fungal species from sequence reads in metagenomics datasets, we first compared the search algorithms BLAST, DIAMOND, Kaiju and Kraken [21, 24–26]. BLAST and DIAMOND both align full reads, whereas Kaiju and Kraken use exact k-mer matches. Kaiju and Kraken map k-mers to the Lowest Common Ancestor (LCA) of all organisms whose genomes contain that k-mer. We tested two versions of Kraken, one with the default k-mer setting of 31 (Kraken 31), and one with a k-mer setting of 16 (Kraken 16).

A test database was constructed from nine bacterial genomes, and one fungal genome. Three simulated metagenomics datasets (Standard, Spiked, and RNA-seq) were generated using Art [31] as shown in Table 1. The Standard dataset was generated from the species in the database. Two additional fungal genomes, and two additional bacterial genomes, not present in the test database, were added to the Spiked dataset. The RNA-seq dataset was generated from only the protein-coding regions from the species from the Standard dataset, and represents a metatranscriptomics experiment. Five tools (BLAST, DIAMOND, Kraken (two versions), and Kaiju [21, 24–26] were tested for their ability to classify reads from the three simulated datasets.

**Table 1:** Species used to generate three simulated read datasets.

Species^1^	Accession Numbers	Number of bp		Simulated dataset (reads)		
		Genome	Exome	Standard	Spiked	RNA-seq
*Bacillus subtilis*	NC_000964.3	4215606	3697728	421560	421560	348870
*Bacteroides fragilis*	NC_006347.1/NC_006297.1	5310990	4787184	531090	531090	455540
*Bifidobacterium bifidum*	NC_014638.1	2214656	1853190	221460	221460	176810
*Lactobacillus acidophilus*	NC_006814.3	1993560	1741788	199350	199350	165210
*Bacillus anthracis*	NC_003997.3	5227293	4234317	522720	522720	397230
*Bartonella henselae*	NC_005956.1	1931047	1386678	193100	193100	131170
*Leptospira borgpetersenii*	NC_008508.1/NC_008509.1	3931782	3023346	393170	393170	285500
*Staphylococcus aureus*	NC_007795.1	2821361	2352093	282104	282110	221610
*Yersinia pestis*	NC_003131.1/NC_003132.1/NC_003134.1/NC_003143.1	4829855	3852405	482980	482980	365300
Candida albicans*	calb_Chr_1 (assembly 19)	3188548	2014897	317216	317172	194026
*Pseudomonas aeruginosa*^§^	NC_002516.2	6264404	-	-	626440	-
*Azotobacter vinelandii* ^§^	NC_012560.1	5365318	-	-	536530	-
Tortispora caseinolytica*^§^	KV453841.1	3117240	-	-	309088	-
Schizosaccharomyces pombe*^§^	NC_003424.3	5579133	-	-	557880	-

^1^Only one chromosome was used from each of the fungal genomes.

*Denotes fungal species.

^§^Denotes species not included in the test database.

The BLAST and Kraken tools were used with databases containing all available nucleotides (‘Genome’, Table 1), whereas the DIAMOND and Kaiju tools were used only with predicted proteins (translated ‘Exome’, Table 1). True positives were defined as reads simulated from a genome that were correctly assigned back to that genome. False positives were defined as reads incorrectly assigned to a genome. True negatives were defined as reads not simulated from a genome that were not assigned to that genome. False negatives were defined as reads simulated from a genome that were not assigned back to that genome. For each method, the sensitivity is defined as the ratio of True Positive to (True Positive + False Negative), and the specificity as the ratio of True Negative to (True Negative + False Positive). Table 2 shows that Kraken 16 displayed the highest sensitivity with all three datasets. However, the specificity is lower than the other methods, especially when used with the Spiked dataset. BLAST and Kraken 31 also had high sensitivity, and higher specificity than Kraken 16 when analyzing the Spiked dataset. DIAMOND and Kaiju both use protein databases, which reduces sensitivity when dealing with untranslatable reads. Kaiju and Kraken were consistently the fastest tools. We chose Kraken 31 to form the basis of the FindFungi pipeline based on its speed, the combination of high sensitivity and specificity, and its ability to assign an LCA prediction to each read.

**Table 2:** Comparison of classification tools using simulated datasets from Table 1.

Dataset	Tool^1^	TP^2^	FP^2^	TN^2^	FN^2^	Sensitivity^3^	Specificity^3^	Time (sec) ^4^
Standard	BLAST	3501029	4	31509237	63779	0.982108714	**0.999999873**	1144.06
Standard	DIAMOND	2625609	5598	23675230	939199	0.736535881	0.999763606	631.34
Standard	Kraken 31	3554377	31	32082661	10431	0.997073896	0.999999034	135.4
Standard	Kraken 16	3563611	41	32082651	1197	**0.999664218**	0.999998722	219.47
Standard	Kaiju	2942976	2332	32080360	621832	0.825563677	0.999927313	**126.2**
RNA-seq	BLAST	2706255	0	24356295	35011	0.987228164	**1**	813.14
RNA-seq	DIAMOND	2537754	120	22840746	203512	0.92575985	0.999994746	497.66
RNA-seq	Kraken 31	2734158	0	24671394	7108	0.997407037	**1**	93.38
RNA-seq	Kraken 16	2741261	2	24671392	5	**0.999998176**	0.999999919	243.92
RNA-seq	Kaiju	2723973	333	24671061	17293	0.993691601	0.999986503	**92.1**
Spiked	BLAST	3501363	2646	31536017	63477	0.982287271	0.999914998	1445.13
Spiked	DIAMOND	2626340	170647	25167565	938500	0.729845366	0.993133657	831.33
Spiked	Kraken 31	3554057	2582	52379078	10783	0.997034142	**0.999950159**	**177.79**
Spiked	Kraken 16	3563615	1288299	51093361	1225	**0.999688747**	0.975408061	424.59
Spiked	Kaiju	2944335	66520	52315140	620505	0.819370262	0.99871138	280.58

^1^For Kraken 31, the test database was divided into 32 individual databases.

^2^Number of reads classified as TP: true positives, FP: false positives, TN: true negatives, FN: false negatives.

^3^sensitivity: TP/(TP + FN), specificity: TN/(TN + FP)

^4^CPU time in seconds. The best sensitivity, specificity, and time for each dataset are highlighted in bold.

### Construction of fungal reference databases

A fungal genome reference database was constructed by downloading all fungal genomes from GenBank. An in-house python script was used to gather all ‘representative’ and ‘reference’ genomes using the GenBank ‘assembly_summary.txt’ file (as of 22-2-17). In total, 949 fungal genomes were collected (32.4 Gb). These genomes were modified to append Kraken taxid (NCBI taxon identification number) identifiers.

To use Kraken, the entire database must be loaded into memory prior to use. However, the storage of 949 fungal genomes in memory is not practical given the memory available on most servers. Therefore, the Kraken database was split into 32 separate databases, and 32 results files were generated for each dataset, using a cluster composed of 32 operational nodes, each with 16 Intel(R) Xeon(R) CPU E5-2670 0 (2.60GHz). To construct the databases, each chromosome/contig in each fungal genome was split into 32 fragments with an overlap of zero, and placed into individual FASTA files. Kraken databases were built from all 32 files. Fungal sequences smaller than 1,100 nucleotides were discarded, amounting to 656 kb or 2% of the total. This conservative cut-off was used to avoid biases from poorly assembled short genomic sequences. In total, the 32 Kraken databases contained 31.8 Gb from the 949 fungal species.

Because 32 different Kraken databases were used in parallel, each read had 32 predictions. These were consolidated using a Python script. The most common prediction was used where possible. If there was no common prediction, the k–mer scoring predictions were concatenated, and the most common k-mer prediction was chosen.

### Using skewness scores to remove false positives

**Fig 1:**
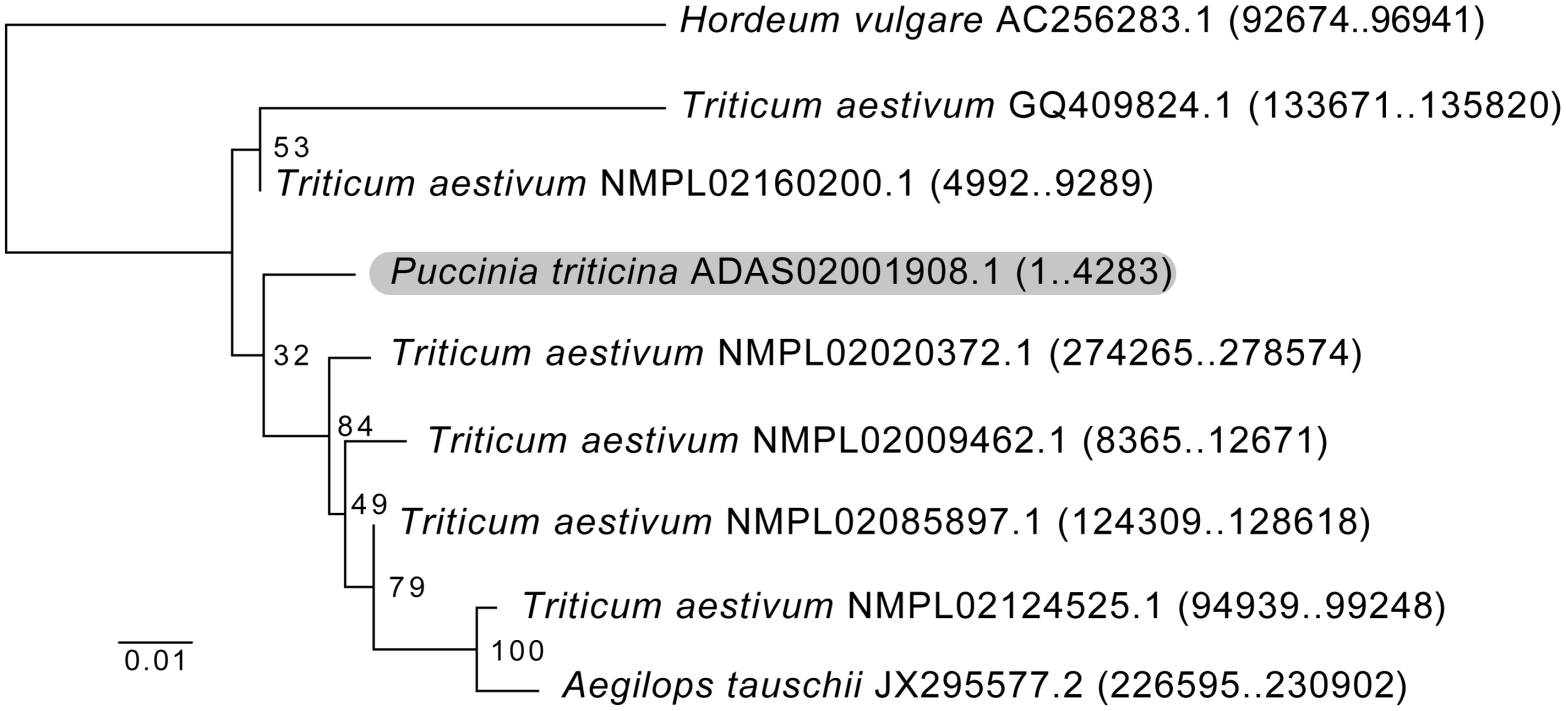
Sequence reads assigned to the fungal pathogen *Puccinia triticina* are derived from a transposable element. Maximum likelihood tree comparing the Copia transposable element from a number of plant genomes and the fungus *P*. *triticina* (shaded). Bootstrap values out of 100 are shown at nodes. Species, chromosome accession, and nucleotide coordinates are displayed. The tree was generated in SeaView using PhyML with the generalized time-reversible (GTR) evolution model using Gblocks and 100 bootstraps.

A preliminary version of the FindFungi pipeline predicted some fungal species in almost all metagenomics datasets, including *Puccina triticina* (the causative agent of wheat leaf rust [32]) and *Talaromyces islandicus* (a mold found on stored rice and cereals [33]). Subsequent analysis showed that these are artifacts, or false positive predictions. For example, BLASTN analysis of a subset of the reads classified as *P*. *triticina* showed that they were derived from a 4,283 bp fungal contig, which matched the wheat genome (*Triticum aestivum*) at 368 different sites, all with at least 92% identity. This sequence is likely to be a Copia transposable element (TE) from *T*. *aestivum* [34] which was incorrectly assembled in the *P*. *triticina* genome (Fig 1).

**Fig 2:**
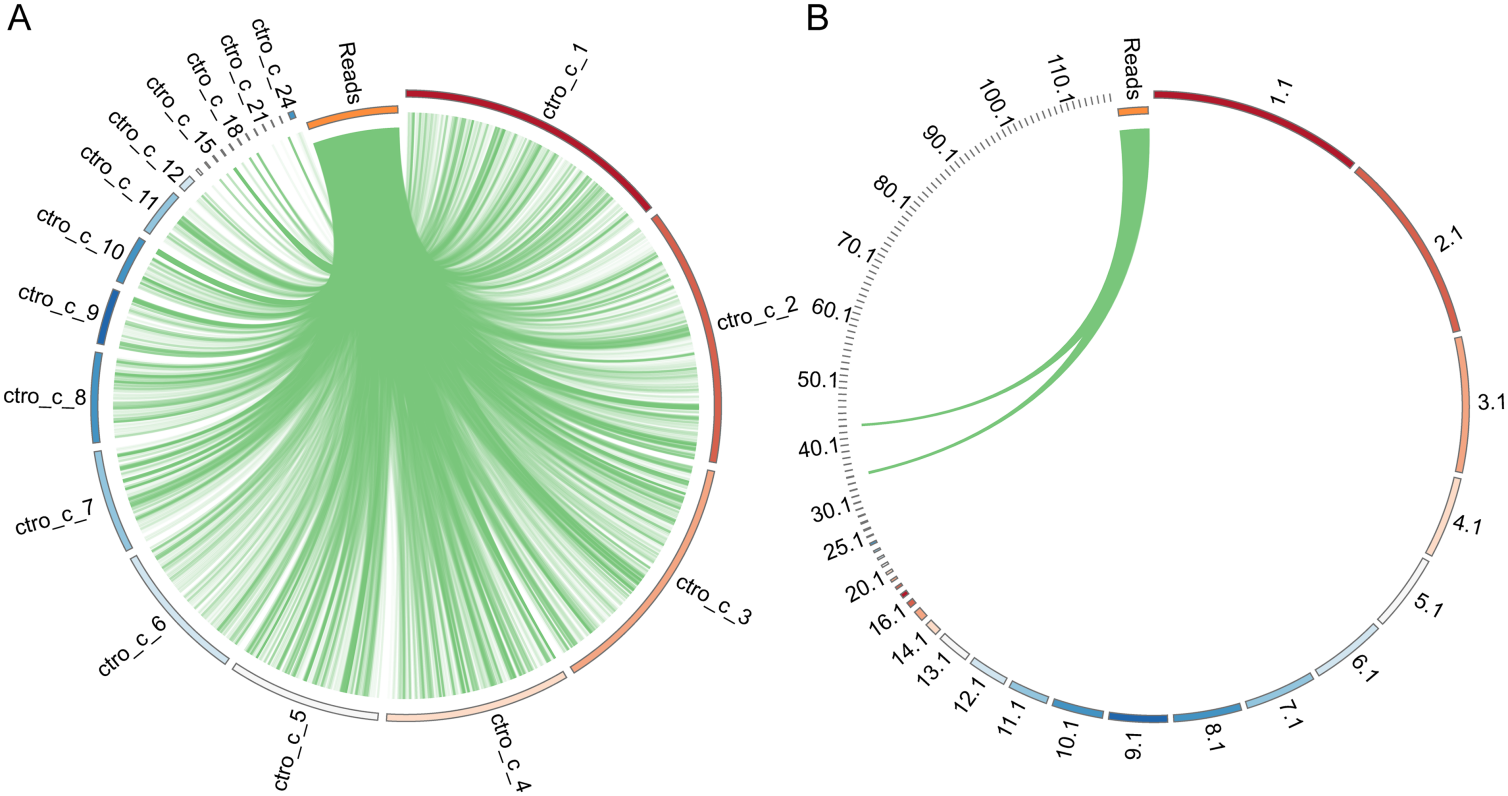
Distinguishing true and false positives using genomic read distribution. (A) Reads classified as *C*. *tropicalis* mapped against the *C*. *tropicalis* MYA-3404 genome. The reads (6,656) were gathered by combining all reads assigned to *C*. *tropicalis* from the datasets ERR675617 and ERR670622. (B) Reads classified as *T*. *islandicus* mapped against the *T*. *islandicus* genome. The reads (7,000) are from the dataset ERR675670. All reads in each analysis were concatenated into a single pseudo-chromosome (orange chromosome with the shortest radius) with 20 ambiguous nucleotides (N) separating each read. The chromosomes in both A and B are colored with a red-to-blue color spectrum. The *T*. *islandicus* label names are abbreviated (e.g. 12.1 displayed instead of CVMT010000012.1). BLAST hits are shown as green links connecting a read with a genomic sequence. The plots were generated using Circos [35].

To address this problem, we examined the distribution of reads from the metagenomics dataset on the genome of the identified species. Reads from a species that is truly present in the dataset are likely to be randomly distributed across the fungal genome, whereas reads from a false positive might show a genomic bias. Fig 2 shows that reads from datasets ERR675617 and ERR670622 that map to *Candida tropicalis* mapped in a random manner across the genome, and likely represent a true positive identification. In contrast, all of the *T*. *islandicus* reads from dataset ERR675670 mapped to two small contigs (CVMT01000034.1 and CVMT01000042.1). Contig CVMT01000034.1 is most similar to the genome of the bacterium *Streptomyces xinghaiensis*, and CVMT01000042.1 to the genome of the bacterium *Lactobacillus gasseri*. It is therefore likely that the *T*. *islandicus* genome assembly contains bacterial contigs.

A read distribution step was therefore incorporated in the FindFungi pipeline. For each of the 949 fungal genomes used to create the Kraken database, all chromosomes/contigs were concatenated into a single super-chromosome, and then divided into 20 pseudo-chromosomes of approximately equal length. BLAST databases were generated for each of these restructured fungal genomes (949 in total). Reads assigned to a particular species by Kraken were compared to the respective BLAST database using an e-value cutoff of 1E-20. The best hit for each read was collected, and the number of reads mapping to each pseudo-chromosome was determined. Pearson’s coefficient of skewness was determined ((mean-median)/standard deviation) using the mean, median, and standard deviation of reads per pseudo-chromosome for each species. The fraction of pseudo-chromosomes that the reads mapped to was also determined.

S1 Fig shows the effect of applying cut-offs based on pseudo-chromosome coverage and skewness score for one dataset, ERR675624. We chose to remove predictions with skewness scores <-0.2 or >+0.2, and reads that mapped to less than 70% of pseudo-chromosomes. Dadi et al [36] also found that determining the distribution of reads from a metagenomics dataset can help to identify false positives. However, some true positives will be lost (S1 Fig), and not all false positives will be removed, particularly those associated with transposable elements or Horizontal Gene Transfer. The cut-offs may therefore be changed to suit different datasets.

**Fig 3:**
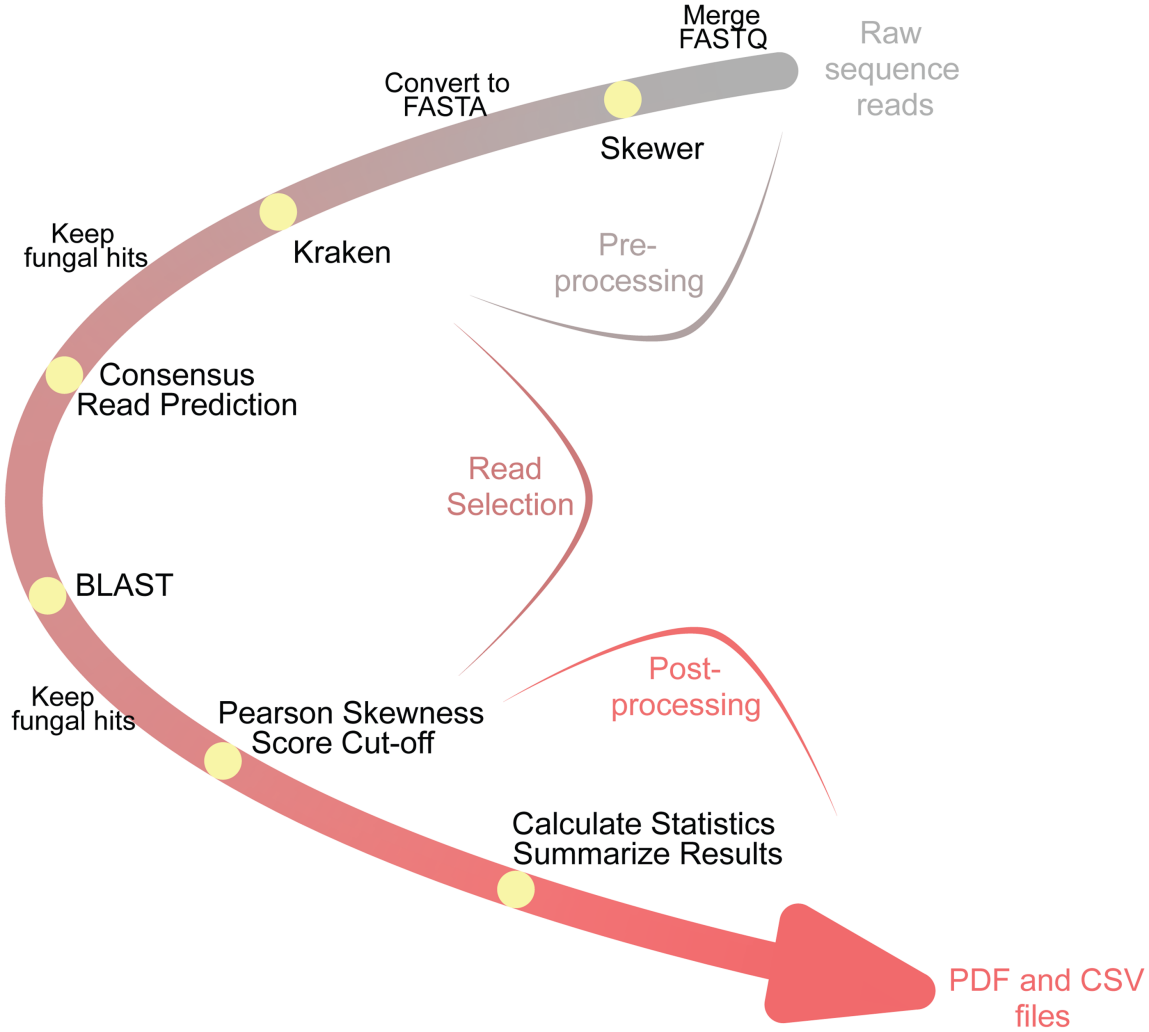
FindFungi v0.23 pipeline overview. Reads are downloaded in FASTQ format. Low quality reads are removed with Skewer [37]. The remaining reads are converted into FASTA format, which are analyzed by 32 implementations of Kraken, each using a different database [26]. The 32 Kraken predictions for each fungal read are consolidated, and a consensus prediction is assigned. Reads not predicted as fungal are removed. The best hit for each read is mapped to a pseudo-assembly of the relevant genome using BLAST [21]. Species where BLAST displays hits on more than 30% of pseudo-chromosomes are retained. Pearson’s coefficient of skewness is calculated to identify non-randomly distributed reads. Species with a skewness score between -0.2 and 0.2 (minimal skew) are retained. Fungal predictions, statistics and summary plots are written to a PDF file, and fungal prediction statistics are also written to a CSV file.

A graphical overview of FindFungi is shown in Fig 3.

### Identification of fungi in metagenomics datasets

The FindFungi v0.23 pipeline was applied to 57 metagenomics datasets from the ‘Host-associated—Mammals’ collection of metagenomics datasets at the EBI Metagenomics database, and 13 additional datasets selected from the MG-RAST database [27]. In total, the 70 datasets contained 2.5 billion reads.

FindFungi predicted the presence of 77 fungal species in 39 datasets (total of 1.2 million fungal reads) (Table 3). To determine if these included any false positive predictions, a subset of the reads predicted for each of the 77 species were compared to the NCBI nt/nr database using BLAST [21]. For six species, read predictions matched bacterial genomes. Manually inspection showed that these reads map to a subset of pseudo-chromosomes. It is likely that these genome assemblies include contaminants (similar to *T*. *islandicus* (Fig 2)), and so the affected species (*Allomyces macrogynus*, *Puccinia arachidis*, *Amauroascus mutatus*, *Amauroascus niger*, *Chryosporium queenslandicum*, *Byssoonygena ceratinophila*) were removed from the predictions (Table 3). The application of Pearson’s coefficient of skewness may therefore not be stringent enough when a very large number of reads are assigned to a species, which should be considered when cut-off limits are assigned.

**Table 3:** Fungal predictions from metagenomics datasets by FindFungi v0.23.

Source	^1^Dataset accession	Total dataset reads	Predicted fungal reads	Fungal predictions (no. of reads)
**Pig microbiome**	ERR1135318	86432970	380	*E*. *bieneusi (213)*, *A*. *brassicae (167)*
**Pig microbiome**	ERR1135427	23597054	491	*R*. *irregularis (413)*, *G*. *luxurians (78)*
**Pig microbiome**	ERR1135453	59108986	1863	*A*. *furcatum (630)*, *P*. *hepiali (575)*, *C*. *militaris (233)*, *B*. *rudraprayagi (161)*, *B*. *bassiana (153)*, *C*. *brongniartii (111)*,
**Pig microbiome**	ERR1135454	30677741	3335	*C*. *confragosa (2574)*, *P*. *hepiali (240)*, *V*. *tricorpus (220)*, *A*. *furcatum (215)*, *B*. *rudraprayagi (86)*
**Pig microbiome**	ERR1135455	57177310	1521	*V*. *tricorpus (581)*, *P*. *hepiali (447)*, *I*. *farinosa (264)*, *C*. *militaris (159)*, *C*. *brongniartii (70)*
**Pig microbiome**	ERR1135750	437278	46	*V*. *tricorpus (46)*
**Pig microbiome**	ERR1223845	62054282	25105	*B*. *anomalus (25105)*
**Vertebrate microbiome**	ERR248260	134577030	35352	*C*. *albicans (26981)*, *D*. *hansenii (2930)*, *D*. *fabryi (1574)*, *M*. *furfur (779)*, *L*. *ramosa (412)*, *T*. *faecale (296)*, *P*. *solitum (281)*, *C*. *sphaerospermum (265)*, *W*. *mellicola (263)*, *T*. *coremiiforme (244)*, *A*. *idahoensis var*. *thermophila (215)*, *U*. *maydis (212)*, *A*. *glaucus (209)*, *M*. *japonica (207)*, *S*. *pastorianus (190)*, *P*. *citrinum (189)*, *P*. *freii (105)*
**Vertebrate microbiome**	ERR248262	141428756	116	*A*. *montevideense (116)*
**Cow microbiome**	ERR571345	5074590	122	*U*. *hordei (122)*
**Mouse microbiome**	ERR675346	731620	6156	*N*. *tetrasperma (5915)*, *N*. *africana (89)*, *N*. *pannonica (85)*, *N*. *terricola (67)*
**Mouse microbiome**	ERR675408	907429	2339	*K*. *phaffii (2047)*, *C*. *gloeosporioides (240)*, *C*. *loboi (52)*
**Mouse microbiome**	ERR675411	809560	2986	*O*. *olearius (2564)*, *U*. *esculenta (422)*
**Mouse microbiome**	ERR675415	857596	88	*C*. *loboi (88)*
**Mouse microbiome**	ERR675422	280130	60	*C*. *loboi (60)*
**Mouse microbiome**	ERR675423	360841	95	*C*. *loboi (95)*
**Mouse microbiome**	ERR675429	511455	95	*C*. *loboi (95)*
**Mouse microbiome**	ERR675603	35832380	57	*R*. *solani (57)*
**Mouse microbiome**	ERR675608	30598678	404	*C*. *loboi (404)*
**Mouse microbiome**	ERR675609	29666898	13451	*C*. *loboi (13109)*, *A*. *domesticum (131)*, *Asp*. *niger (85)*, *C*. *sojae (72)*, *R*. *solani (54)*
**Mouse microbiome**	ERR675612	3883030	2314	*C*. *loboi (1599)*, *C*. *tropicalis (715)*
**Mouse microbiome**	ERR675617	27007988	11589	*C*. *loboi (7703)*, *C*. *tropicalis (3675)*, *A*. *domesticum (118)*, *R*. *solani (93)*
**Mouse microbiome**	ERR675618	27288536	341	*C*. *loboi (341)*
**Mouse microbiome**	ERR675622	23395904	9753	*C*. *loboi (6611)*, *C*. *tropicalis (2981)*, *A*. *domesticum (93)*, *R*. *solani (68)*
**Mouse microbiome**	ERR675624	16893482	1314	*C*. *loboi (671)*, *M*. *restricta (378)*, *C*. *tropicalis (265)*
**Mouse microbiome**	ERR675626	21805514	910	*C*. *loboi (910)*
**Antarctic soil**	mgm4721951.3	1726909	157390	*P*. *sp*. *VKMF-4515 (96310)*, *P*. *sp*. *VKMF-4517 (41360)*, *P*. *destructans (12367)*, *P*. *sp*. *VKMF-3808 (2760)*, *P*. *sp*. *24MN13 (2338)*, *C*. *confragosa (1823)*, *P*. *arachidis (457)*, *I*. *farinosa (105)*, *C*. *militaris (92)*, *B*. *rudraprayagi (81)*, *C*. *herbarum (78)*, *C*. *brongniartii (76)*
**Antarctic soil**	mgm4721952.3	2867433	411	*M*. *alpina (173)*, *P*. *sp*. *VKM F-4281 (124)*, *P*. *sp*. *VKM F-4518 (114)*
**Antarctic soil**	mgm4721953.3	2119288	229853	*P*. *sp*. *VKM F-4515 (141981)*, *P*. *sp*. *VKM F-4517 (54195)*, *P*. *sp*. *VKM F-4518 (18787)*, *P*. *sp*. *BL308 (11409)*, *P*. *sp*. *24MN13 (2874)*, *C*. *confragosa (331)*, *C*. *herbarum (186)*, *P*. *hepiali (90)*
**Antarctic soil**	mgm4721954.3	3215171	412	*P*. *sp*. *VKM F-4520 (196)*, *P*. *sp*. *VKM F-4515 (148)*, *P*. *destructans (68)*
**Antarctic soil**	mgm4721955.3	1105951	1558	*P*. *sp*. *VKM F-4515 (543)*, *P*. *sp*. *VKM F-4517 (403)*, *P*. *sp*. *VKM F-4281 (290)*, *C*. *confragosa (223)*, *P*. *hepiali (54)*, *P*. *sp*. *BL308 (45)*
**Antarctic soil**	mgm4721956.3	1097260	263	*P*. *sp*. *VKM F-4281 (129)*, *P*. *sp*. *VKM F-4515 (90)*, *P*. *sp*. *VKM F-4520 (44)*
**Antarctic soil**	mgm4721957.3	2059400	27267	*P*. *sp*. *VKM F-4515 (14221)*, *P*. *sp*. *VKM F-4517 (9269)*, *P*. *destructans (1337)*, *C*. *confragosa (1144)*, *P*. *sp*. *VKM F-3808 (450)*, *P*. *sp*. *24MN13 (374)*, *P*. *sp*. *VKM F-103 (195)*, *I*. *fumosorosea (91)*, *B*. *rudraprayagi (68)*, *M*. *guizhouense (68)*, *P*. *subalpina (50)*
**Antarctic soil**	mgm4721958.3	1294113	1364	*P*. *sp*. *VKM F-4515 (553)*, *P*. *sp*. *VKM F-4581 (329)*, *P*. *sp*. *VKM F-4517 (270)*, *P*. *sp*. *VKM F-4518 (116)*, *P*. *sp*. *VKM F-4520 (96)*
**Antarctic soil**	mgm4721959.3	358379	190	*P*. *sp*. *VKM F-4515 (142)*, *M*. *alpina (48)*
**Antarctic soil**	mgm4721960.3	1067649	5899	*P*. *sp*. *VKM F-4517 (3927)*, *P*. *sp*. *VKM F-4518 (534)*, *P*. *sp*. *BL308 (481)*, *P*. *destructans (312)*, *P*. *sp*. *VKM F-3775(172)*, *P*. *sp*. *04NY16 (134)*, *P*. *verrucosus (107)*, *P*. *pannorum var*. *pannorum (99)*, *P*. *sp*. *VKM F-4246(67)*, *P*. *sp*. *VKM F-4514 (66)*
**Antarctic soil**	mgm4721961.3	1686048	28885	*P*. *sp*. *VKM F-4517 (24109)*, *P*. *sp*. *BL308 (1449)*, *P*. *sp*. *VKM F-4518 (1017)*, *P*. *sp*. *VKM F-4520 (911)*, *P*. *sp*. *VKM F-3775 (409)*, *P*. *sp*. *24MN13 (306)*, *P*. *sp*. *VKM F-3808 (266)*, *M*. *alpina (195)*, *P*. *pannorum (157)*, *P*. *sp*. *BL549 (66)*
**Antarctic soil**	mgm4721962.3	2063872	6260	*P*. *sp*. *VKM F-4517 (2665)*, *P*. *sp*. *VKM F-4581 (2181)*, *P*. *sp*. *VKM F-4518 (504)*, *P*. *sp*. *BL308 (283)*, *P*. *sp*. *24MN13 (204)*, *P*. *sp*. *VKM F-3775 (142)*, *P*. *sp*. *04NY16 (119)*, *P*. *sp*. *VKM F-3808 (103)*, *P*. *sp*. *VKM F-103 (59)*
**Antarctic soil**	mgm4721963.3	2287098	633283	*P*. *sp*. *VKM F-4281 (472319)*, *P*. *sp*. *VKM F-4517 (86947)*, *P*. *destructans (30245)*, *A*. *sp*. *Z5 (21574)*, *P*. *sp*. *BL308 (14361)*, *P*. *sp*. *24MN13 (6000)*, *C*. *confragosa (1427)*, *C*. *herbarum (276)*, *I*. *farinosa (77)*, *I*. *fumosorosea (57)*
-	**70 datasets**	844345609	1213318	**-**

^1^ERR1135227, ERR1135237, ERR1135245, ERR1135256, ERR1135268, ERR1135269, ERR1135291, ERR1135346, ERR1135368, ERR1135372, ERR1135406, ERR1135418, ERR1135429, ERR1135449, ERR1135459, ERR1135749, ERR1223846, ERR675430, ERR675519, ERR675529, ERR675568, ERR675616, ERR675632, ERR675653, ERR675654, ERR675670, ERR675674, ERR675677, ERR675680, ERR675682, ERR675683 had no fungal reads.

### Identification of *Pseudogymnoascus* species in Antarctic soils

A group of 13 MG-RAST datasets came from a project analyzing the role of bacteria in diesel-oil biodegradation in Antarctic soil, and were predicted by MG-RAST to contain fungal species (Table 3). The FindFungi pipeline classified 4.91% of the reads (>1 million reads) from all of these datasets as originating from the *Pseudogymnoascus* (*Geomyces*) genus. *Pseudogymnoascus* species are psychrotolerant (cold-tolerant) [38], and some species have previously been isolated from Antarctic soils [38, 39]. *Pseudogymnoascus pannorum*, which was found in two datasets, has been linked to the biodegradation of diesel-oil in the Amazon [40]. Therefore, it is possible that the *Pseudogymnoascus* species identified in the Antarctic diesel-oil study are responsible, at least in part, for the biodegradation of the diesel-oil. FindFungi identified *Pseudogymnoascus destructans* in five of the 13 Antarctic diesel-oil datasets (Table 3). *P*. *destructans* is a true psychrophilic (cold-loving) species, and is the causative agent of the disease known as White-Nose Syndrome that is decimating bat populations in the US [38].

### Identification of potentially pathogenic fungi

**Fig 4:**
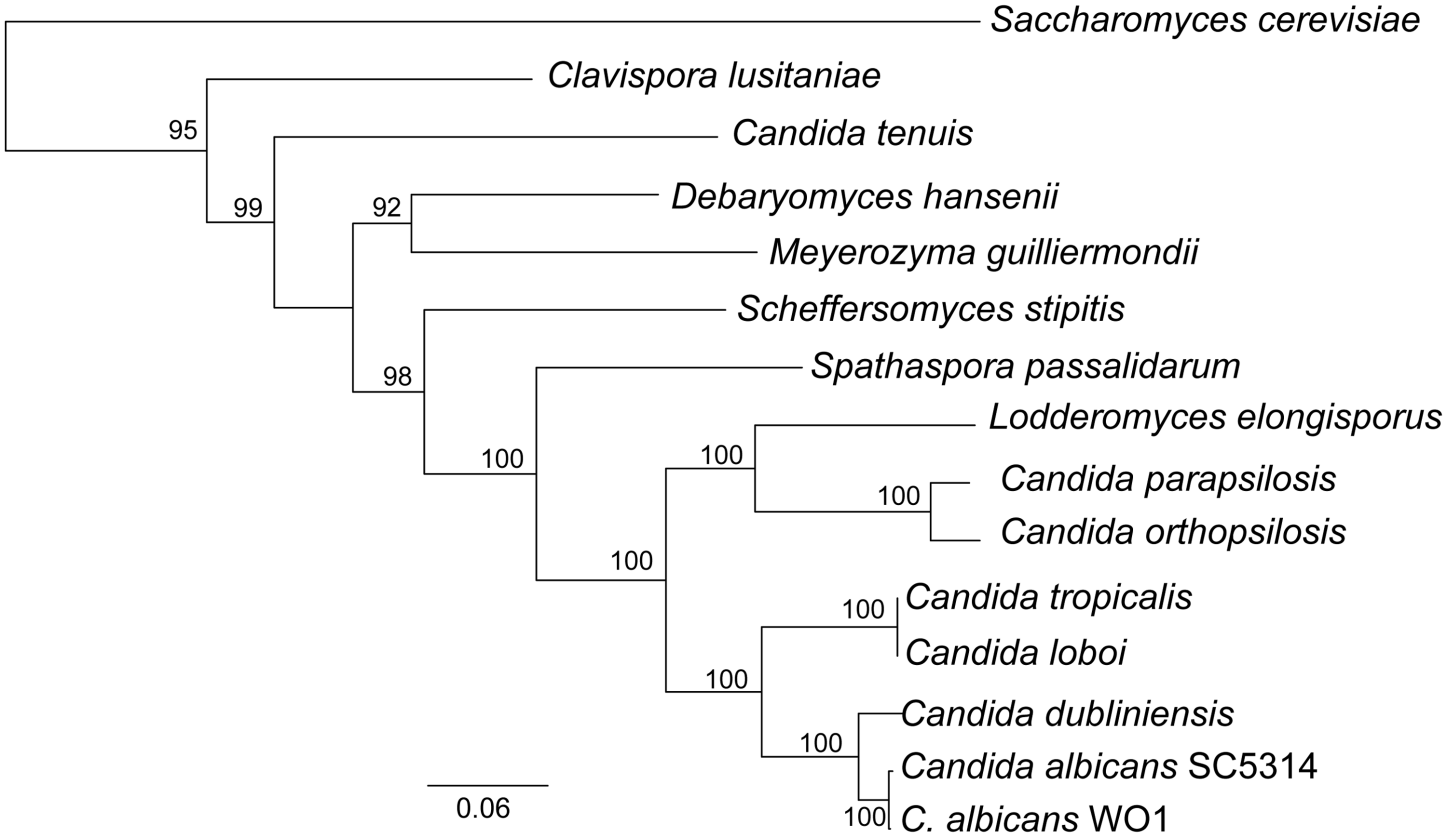
*Candida loboi* and *Candida tropicalis* are isolates of the same species. Maximum likelihood tree of a concatenated five-protein alignment from species from the *Candida* Gene Order Browser (CGOB; [46]) and *C*. *loboi*. Five genes (*ERG1*, *MEF1*, *CEF3*, *DEG1*, *GCD14*) that are conserved in all CGOB species were chosen at random. All *C*. *loboi* orthologs were identified with best BLAST matches using *C*. *tropicalis* gene homologs. Protein sequences were aligned using Muscle (v3.8.31, [47]) and concatenated. The tree was generated in SeaView [48] using PhyML with the LG evolution model using Gblocks [49] and 100 bootstraps (shown at nodes). Species abbreviations are displayed at branch leaves.

FindFungi identified reads from human fungal pathogens, particularly *Candida* species, in 16 datasets (Table 3). *Candida albicans*, the most prevalent *Candida* species in human fungal infections [41] was identified in only one dataset (ERR248260, Table 3) from an unidentified vertebrate mammal. However, FindFungi assigned > 31,000 reads to *Candida sp*. *LDI48194*, also known as *Lacazia loboi* [42] from 13 datasets from the Mouse Gut Metagenome Project (ERP008710). *L*. *loboi* is a poorly characterized causative agent of lobomycosis, and has been associated with pathogenicity in both humans and dolphins with zoonotic potential [43]. Up until 2015, this species was classified as a member of the genus *Lacazia*. However, following genome sequencing, it was reclassified as *Candida loboi*, part of the CTG-Ser clade. FindFungi also predicted *Candida tropicalis* in four of the datasets containing *C*. *loboi* (Table 3). *C*. *tropicalis* is an emerging human fungal pathogen that has previously been identified in the microbiomes of mice, where they may be endogenous species [44, 45]. We examined the relationship between *C*. *tropicalis* and *C*. *loboi* using phylogenetic analysis based on a concatenated alignment of five proteins (Fig 4). The *C*. *loboi* and *C*. *tropicalis* proteins are more similar to each other (99.9% identity) than proteins from two *C*. *albicans* isolates (SC5314 and WO1, 99.6% identity), strongly suggesting that they are both isolates of the same species.

Human fungal pathogens associated with less-severe disease states were also identified, including members of the *Malassezia* and *Enterocytozoon* species families. *Malassezia restricta* was discovered in one dataset, and the related species *Malassezia furfur* and *Malassezia japonica* were discovered in a second (Table 3). These species are responsible for a number of hair and skin infections such as seborrheic dermatitis [50]. *Enterocytozoon bieneusi*, a Microsporidia species that infects intestinal epithelial cells, was identified in a pig microbiome dataset (Table 3). This species is associated with infection in both humans and animals. Pigs with *E*. *bieneusi* in their gut are generally asymptomatic and are therefore not treated, permitting dissemination of the pathogen both throughout swine herds and across the species-barrier to humans [51]. Pigs represent the main animal reservoir of *E*. *bieneusi* [52]. From a human perspective, *E*. *bieneusi* is an emerging pathogen that primarily infects immunocompromised individuals and can cause life-threatening diarrhea [51].

The Pezizomycotina fungus *Cladosporium sphaerospermum* was identified in an unknown vertebrate microbiome (Table 3). This species has been associated with respiratory infections and is a major allergen [53]. *Trichosporon coremiiforme* was identified in the same dataset. Although generally considered as a human commensal, this species has also been shown to grow as a biofilm and to evade common antifungals [54]. *Apiotrichum montevideense* is a member of the Basidiomycota, and is a close relative of *Cryptococcus* and *Trichosporon* species. *A*. *montevideense* is one of the causative agents of summer-type hypersensitivity pneumonitis [55], and was identified in a different unknown vertebrate microbiome (Table 3). *Apiotrichum domesticum*, which causes the same disease [55], was identified in three mouse microbiomes (Table 3). FindFungi did not identify animal reservoirs for other significant human fungal pathogens such as *Cryptococcus neoformans*, *Pneumocystis jirovecii*, *Coccidioides immitis*, *Histoplasma capsulatum*, or *Trichophyton rubrum*.

### Identification of fungi not pathogenic to humans

Several insect pathogens were identified in the animal microbiome datasets. 2,574 reads from the insect parasite *Cordyceps confragosa* [56] were identified in a pig microbiome (ERR1135454, Table 3). 153 reads from the related species *Beauveria bassiana* [57], were discovered in a second dataset (ERR1135453, Table 3). Other species from the Cordycipitaceae family (including *Isaria*, *Cordyceps*, and *Beauveria* species) were also identified (ERR1135453 –ERR1135455, Table 3). *Acremonium furcatum*, a member of a fungal family that produces cephalosporins [58] was identified in two microbiomes from pig stools (Table 3). Another insect pathogen, *Metarhizium guizhouense* [59], was identified in an Antarctic soil sample (mgm4721957.3, Table 3).

Fungal plant pathogens were also identified. *Aspergillus niger*, the causative agent of black mold on fruits and vegetables [60], was found in a mouse microbiome (ERR675609, Table 3). 122 reads from a bovine feces sample (ERR571345, Table 3), were predicted to originate from *Ustilago hordei*, a barley fungal pathogen [61]. The related grain pathogens [62] *Ustilago esculenta* and *Ustilago maydis* were found in a mouse microbiome (ERR675411, Table 3) and an unknown vertebrate microbiome (ERR248260, Table 3), respectively. A number of other plant pathogens were identified, including *Verticillium tricorpus* (opportunistic plant pathogen [63]), *Colletotrichum gloeosporioides* [64], *Phialocephala subalpina* [65], and *Rhizoctonia solani* [66]. We do not know the origins of the plant pathogens, but they may originate from feed or bedding materials.

Species associated with industrial applications such as *Komagataella phaffii* (*Pichia pastoris*), a methylotroph used for protein production [67] and *Brettanomyces anomalus*, a yeast typically associated with beer and wine fermentation [68], were identified in a mouse microbiome (ERR675408) and from the floor of a pigpen (ERR1223845), respectively (Table 3).

## Conclusion

The decrease in sequencing costs and improvements in sequencing technology has resulted in a dramatic increase in the availability of sequencing data over the past decade. Culture-free shotgun metagenomics sequencing is becoming a popular strategy for various analyses, and may replace ITS or barcode sequencing. Much of these data are generated for a specific purpose, and are then deposited in a database such as the Sequence Read Archive, with no intention of further use.

We have shown that FindFungi can be used to identify fungi from publicly available shotgun metagenomics datasets. We focused our analyses on 57 animal shotgun metagenomics datasets from the EBI-Metagenomics database and 13 MG-RAST datasets. FindFungi predicted fungal DNA in 39 of the analyzed datasets. We identified potential zoonotic fungi in animal microbiomes, and a large number of psychrophilic fungi in Antarctic soil. We showed that several fungal genomes have assembly errors, including bacterial contamination. FindFungi can be applied to any shotgun metagenomics dataset.

## Supporting information

S1 Fig Evaluation of cut-offs for FindFungi species identification. Species identified by FindFungi from dataset ERR675624 before cut-offs were applied were categorized as true positives (TP, blue) or false positives (FP, red) by comparing 10 randomly selected reads from each species prediction against the NCBI nt/nr database (BLASTn and BLASTx). Reads that supported the FindFungi prediction (same species or a close relative), were deemed to be true positives. The boxed region shows skewness cut-offs range from -0.2 to 0.2 and chromosome coverage cut-off ranges from 70–100%. These cut-offs were applied to subsequent predictions by FindFungi.
(PDF)
