# Supplementary material for: Identification of fungi in shotgun metagenomics datasets
Source: PLoS One. 2018 Feb 14;13(2):e0192898. doi: 10.1371/journal.pone.0192898 (PMC5812651; doi:10.1371/journal.pone.0192898)
Supplement: S1 Fig — Species identified by FindFungi from dataset ERR675624 before cut-offs were applied were categorized as true positives (TP, blue) or false positives (FP, red) by comparing 10 randomly selected reads from each species prediction against the NCBI nt/nr database (BLASTn and BLASTx). Reads that supported the FindFungi prediction (same species or a close relative), were deemed to be true positives. The boxed region shows skewness cut-offs range from -0.2 to 0.2 and chromosome coverage cut-off ranges from 70–100%. These cut-offs were applied to subsequent predictions by FindFungi. (PDF) [file pone.0192898.s001.pdf]

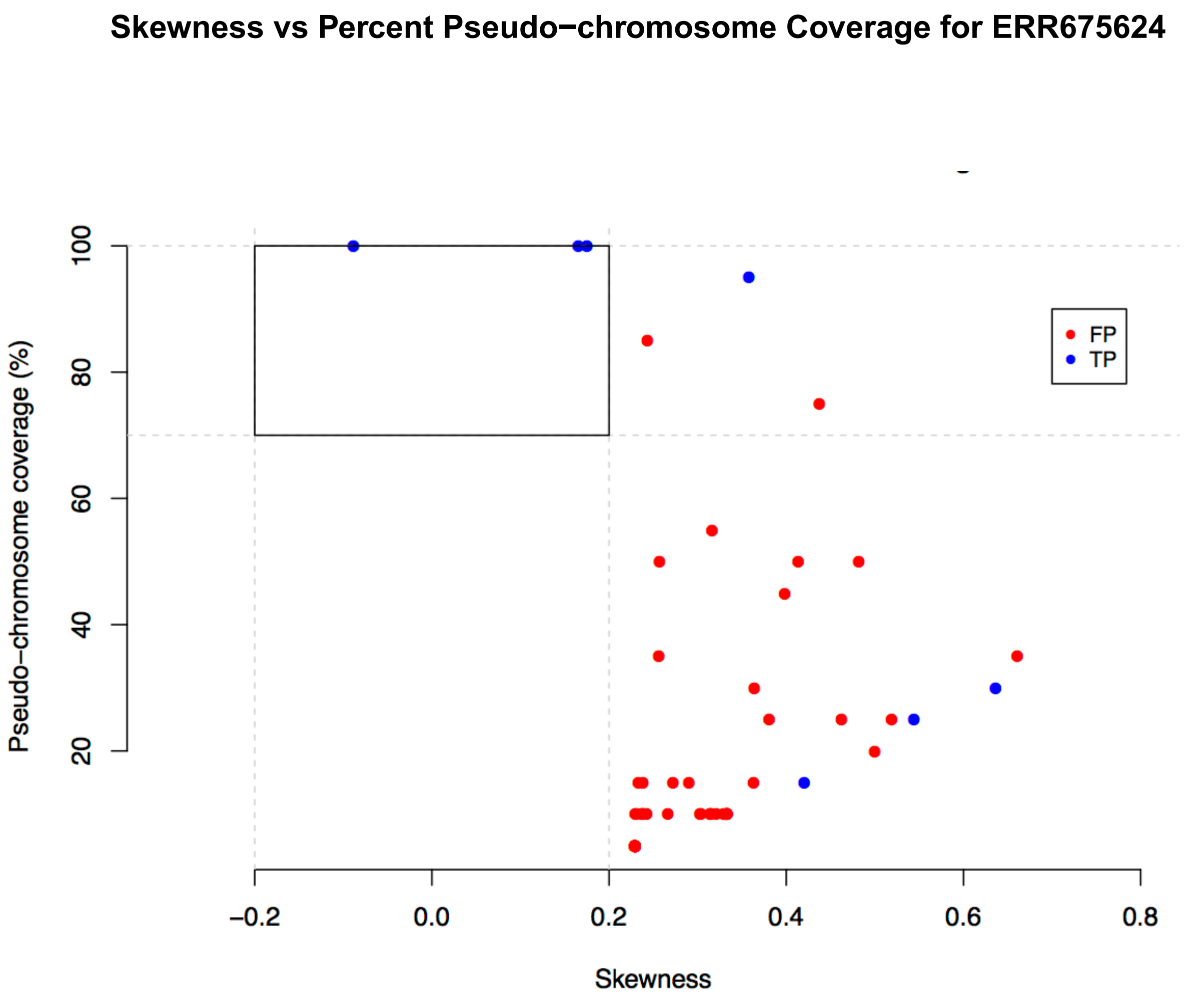

S1 Fig. Evaluation of cut-offs for FindFungi species identification.

Species identified by FindFungi from dataset ERR675624 before cut-offs were applied were categorized as true positives (TP, blue) or false positives (FP, red) by comparing 10 randomly selected reads from each species prediction against the NCBI nt/nr database (BLASTn and BLASTx). Reads that supported the FindFungi prediction (same species or a close relative), were deemed to be true positives. The boxed region shows skewness cut-offs range from -0.2 to 0.2 and chromosome coverage cut-off ranges from 70-100%. These cut-offs were applied to subsequent predictions by FindFungi.
